# Microstructural Evolution of Dy_2_O_3_-TiO_2_ Powder Mixtures during Ball Milling and Post-Milled Annealing

**DOI:** 10.3390/ma10010019

**Published:** 2016-12-28

**Authors:** Jinhua Huang, Guang Ran, Jianxin Lin, Qiang Shen, Penghui Lei, Xina Wang, Ning Li

**Affiliations:** College of Energy, Xiamen University, Xiamen 361102, China; jhhuang@stu.xmu.edu.cn (J.H.); jxlin@stu.xmu.edu.cn (J.L.); shenqiang1989@126.com (Q.S.); p.h.lei@foxmail.com (P.L.); xina_wang@126.com (X.W.); Ningli@xmu.edu.cn (N.L.)

**Keywords:** microstructure, ball milling, dysprosium oxide, neutron absorber, phase evolution

## Abstract

The microstructural evolution of Dy_2_O_3_-TiO_2_ powder mixtures during ball milling and post-milled annealing was investigated using XRD, SEM, TEM, and DSC. At high ball-milling rotation speeds, the mixtures were fined, homogenized, nanocrystallized, and later completely amorphized, and the transformation of Dy_2_O_3_ from the cubic to the monoclinic crystal structure was observed. The amorphous transformation resulted from monoclinic Dy_2_O_3_, not from cubic Dy_2_O_3_. However, at low ball-milling rotation speeds, the mixtures were only fined and homogenized. An intermediate phase with a similar crystal structure to that of cubic Dy_2_TiO_5_ was detected in the amorphous mixtures annealed from 800 to 1000 °C, which was a metastable phase that transformed to orthorhombic Dy_2_TiO_5_ when the annealing temperature was above 1050 °C. However, at the same annealing temperatures, pyrochlore Dy_2_Ti_2_O_7_ initially formed and subsequently reacted with the remaining Dy_2_O_3_ to form orthorhombic Dy_2_TiO_5_ in the homogenous mixtures. The evolutionary mechanism of powder mixtures during ball milling and subsequent annealing was analyzed.

## 1. Introduction

High-energy ball milling has been widely used to prepare various types of materials, such as supersaturated solid solutions, metastable crystalline materials [1], quasicrystal phases [2], nanostructured materials [3], and amorphous alloys [4]. The technology was initially used in place of blending and sintering at elevated temperatures to prepare ceramic-strengthened alloys [5,6]. A large amount of mechanical energy is transformed into intrinsic energy in the target materials, which induces the formation of numerous defects in the crystal structure, such as vacancies, interstitials, cavities and dislocations, which are always in a non-equilibrium state [7,8,9]. The defects and structural disorders will increase the mobility of atomic diffusion and induce chemical reactions amongst components that are not present under equilibrium conditions [10]. Therefore, based on its excellent characteristics, ball milling was used to prepare bulk Dy_2_TiO_5_, which can be used as a neutron absorber in control rods in nuclear power plants. Control rods are very important in both operating and accident conditions because the nucleon reactivity must be controlled in order to safely operate a nuclear reactor [11]. In fact, bulk Dy_2_TiO_5_ prepared by ball milling and sintering has been used in Russian power plant water reactors, such as MIR and VVER-1000 RCCAs [12,13], because of the excellent nucleon characteristics of the element dysprosium, as natural dysprosium consists of five stable isotopes with high thermal neutron absorption cross sections. The decay products are Ho and Er, which are also able to absorb neutrons. All of the radionuclides have low gamma activity and short half-life periods. The absorption cross sections of dysprosium isotopes range from 130 barn to 2600 barn. The region of resonance absorption is 1.6–25 eV, in which the absorption cross-section can reach approximately 1000 barn [14].

According to its equilibrium phase diagram, Dy_2_TiO_5_ has three crystal structural types depending on the temperature, orthorhombic $\overset{1350\;{}^{\circ}\text{C}}{↔}$ hexagonal $\overset{1680\;{}^{\circ}\text{C}}{↔}$ cubic [15], which have different physical properties and radiation resistance abilities. In fact, bulk Dy_2_TiO_5_ in the cubic crystal structure has the lowest neutron irradiation swelling and highest irradiation resistance. Therefore, it is necessary to synthesize bulk Dy_2_TiO_5_ in the cubic crystal structure. Jung [16] synthesized bulk Dy_2_TiO_5_ with high purity and density using a polymer carrier chemical synthesis process, in which ethylene glycol was used as an organic carrier for metal cations. An amorphous phase was detected below 800 °C, and orthorhombic Dy_2_TiO_5_ was observed after sintering for 1 h at 1300 °C, while little else was observed while sintering in the range of 800 to 1300 °C. Panneerselvam [17] used both solid-state synthesis and wet chemical synthesis to prepare Dy_2_TiO_5_. However, the effect of sintering temperature on the phase evolution needs further investigation. The sinterability of Dy_2_O_3_ and TiO_2_ with different molar ratios was determined for various ball-milling and sintering conditions [18]. Amit Sinha [19] reported the synthesis of bulk Dy_2_TiO_5_ from mixtures of equimolar Dy_2_O_3_ and TiO_2_ powders in a two-step process: (I) pyrochlore Dy_2_Ti_2_O_7_ was initially formed and (II) Dy_2_Ti_2_O_7_ then reacted with the remaining Dy_2_O_3_ to form orthorhombic Dy_2_TiO_5_. The powder mixtures used in sintering were simply mixed during ball milling. Garcia-Martinez [7] observed the experimental phenomena of the transformation of Dy_2_O_3_ from cubic to monoclinic and the synthesis of a hexagonal high-temperature phase, reported as Dy_2_TiO_5_, in an equimolar Dy_2_O_3_-TiO_2_ mixture during ball milling. Therefore, further investigation is needed into the evolutionary behavior of the microstructure under different ball-milling conditions and the effect of the state of the ball-milled powder on the sintering behavior. In the present work, the microstructural evolutionary behavior and corresponding reaction mechanism of Dy_2_O_3_-TiO_2_ powder mixtures under two types of ball-milling parameters were investigated. The annealing behavior of the ball-milled mixtures was also examined.

## 2. Experiments

Powders of Dy_2_O_3_ (cubic crystal structure) and TiO_2_ (rutile crystal structure) with an average particle diameter of 5 μm and 50 nm, respectively, were used as raw materials. The raw powders of Dy_2_O_3_ and TiO_2_ were purchased from Beijing HWRK Chem Co., Ltd. (Beijing, China). The purity of the raw powders of both Dy_2_O_3_ and TiO_2_ was 99.9%. Ball milling of the molar fraction Dy_2_O_3_-50% TiO_2_ (Dy_2_O_3_:TiO_2_ = 1:1) powder mixtures was carried out on an SFM-1 high-energy planetary ball mill at room temperature. Stainless steel balls that were 5 mm in diameter were used as the milling media. The ball-to-powder mass ratio was 10:1, and the rotational speed was 200 rpm and 500 rpm. No more than one weight percent stearic acid was added to the powder mixtures as a process control agent to prevent excessive cold welding and aggregation amongst the powder particles. During ball milling, a 5-min stopping interval was used after milling for 55 min to prevent excess heat generation, which has an obvious effect on the ball-milling procedure. The powder mixtures used for microstructural analysis were extracted from the loose powders in the steel can, not from powders adhered to the surface of the stainless balls or the steel can wall, after ball milling for 4, 12, 24, 48, and 96 h.

After various milling times, a small amount of ball-milled powders taken from the container were characterized and analyzed by X-ray diffraction (XRD) on a Rigaku Ultima IV X-ray diffractometer (Rigaku, Tokyo, Japan) with Cu Kα radiation (λ = 0.1540598 nm) and transmission electron microscopy (TEM) on a JEM-2100 instrument (JEOL, Tokyo, Japan). Analysis was also carried out for ball-milled powder mixtures annealed at different temperatures.

The grain size was calculated using Suryanarayana and Grant Norton’s formula [20].

(1) $$B_{r}\cos\theta \;=\;\frac{K\lambda }{L}\;+\;\eta \sin\theta$$

where, K is a constant (with a value of 0.9); *λ* is the wavelength of the X-ray radiation; *L* and η are the grain size and internal strain, respectively; and θ is the Bragg angle. *B_r_* is the full width at half-maximum (FWHM) of the diffraction peak after instrumental correction and can be calculated from the following equation:

*B* = *B_r_* + *B_s_* (2)

where, *B* and *B_S_* are the FWHM of the broadened Bragg peaks and the standard sample’s Bragg peaks, respectively.

The ball-milled mixtures and annealed mixtures were first put in ethyl alcohol, and then adequately dispersed by ultrasonic vibration. A carbon-coated copper grid was used to collect the dispersed powders in the ethyl alcohol and then dried by ultraviolet lamp. After that, the prepared samples were observed by TEM. Differential scanning calorimetry (DSC) was used to analyze the thermal behavior of ball-milled powders at a 5 °C/min heating rate in argon atmosphere using a SAT 449C instrument (NETZSCH, Bavarian State, Germany ). The powder mixtures milled for 96 h were annealed at temperatures ranging from 700 to 1150 °C in a tube furnace under atmospheric conditions. The heating and cooling rates were both 5 °C/min.

## 3. Results and Discussion

**Figure 1:**
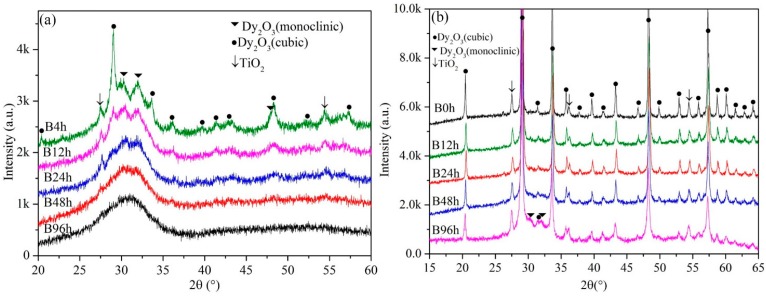
X-ray diffraction patterns of the powder mixtures milled at (**a**) 500 rpm and (**b**) 200 rpm for various times, respectively.

The XRD patterns of Dy_2_O_3_-TiO_2_ powder mixtures milled at 500 rpm and 200 rpm for different times are shown in Figure 1. The XRD results show that the crystal structure of the original Dy_2_O_3_ phase and TiO_2_ phase are cubic and rutile, respectively. At the condition of 500 rpm, the diffraction peaks of cubic Dy_2_O_3_ and TiO_2_ broadened significantly and reduced in intensity with increased milling time. The broadening of the X-ray diffraction peaks is associated with the refinement in grain size and lattice distortions. Meanwhile, the diffraction peaks of monoclinic Dy_2_O_3_ can be observed in the X-ray patterns as indicated by the black inverted triangles in Figure 1a. Ball milling induces a Dy_2_O_3_ phase transformation from the cubic to the monoclinic crystal structure. A broad, singular diffraction peak is also present that indicates the formation of the amorphous phase during ball milling. Interestingly, the amorphous peak is present at the location of the diffraction peak for the monoclinic Dy_2_O_3_ phase, not at the location of the diffraction peak for the cubic Dy_2_O_3_ phase, which indicates that the formed amorphous phase is derived from the monoclinic Dy_2_O_3_ phase, not from the cubic Dy_2_O_3_ phase. The transformation from cubic to monoclinic increases with increased milling time. After milling for 96 h, only the amorphous phase can be observed, which indicates that the monoclinic Dy_2_O_3_ phase was fully converted to the amorphous phase. Additionally, this behavior indicates that no new compounds are synthesized during ball milling. Even if new compounds were formed in the milled powders, the amount is very low and does not reach the sensitivity range of the X-ray measurement. Therefore, in the present work, the evolution of Dy_2_O_3_-TiO_2_ powder mixtures is as follows: ball milling first induces the transformation of Dy_2_O_3_ from the cubic to the monoclinic crystal phase, then monoclinic Dy_2_O_3_ undergoes amorphization, and finally the powder mixtures completely transform to the amorphous phase.

However, the ball-milling behavior of powder mixtures at 200 rpm is distinctly different from that at 500 rpm. The change of the diffraction peaks with increased milling time at 200 rpm is shown in Figure 1b. Although the diffraction peaks of cubic Dy_2_O_3_ and TiO_2_ are also broadened and reduced in intensity with increased milling time, the diffraction peaks of TiO_2_ can be observed in the XRD spectrums and are not disappeared. After ball milling for 96 h, the intensity of diffraction peaks of Dy_2_O_3_ and TiO_2_ are also high. The powder mixtures are not changed completely to amorphization. According to the shape of XRD diffraction spectrums, the effect of ball milling on powder mixtures after milling for 96 h at 200 rpm is only similar to that after milling for 4 h at 500 rpm. Therefore, at low ball-milling rotation speeds, the powder mixtures are only fined and homogenized.

Our experimental results are different from the results of G. Garcia-Martinez [7]. In their research, ball milling induced a phase transformation in Dy_2_O_3_ from cubic to monoclinic. However, a Dy_2_TiO_5_ compound with a hexagonal crystal structure was formed simultaneously. The ball-milled powders consisted of mixed phases of hexagonal Dy_2_TiO_5_ and monoclinic Dy_2_O_3_. The Dy_2_O_3_-TiO_2_ powder mixtures did not completely transform to the amorphous phase, but instead produced the hexagonal Dy_2_TiO_5_ phase. This difference can be attributed to the different ball-milling conditions used in this research, with special attention to the different ball-milling facilities. During ball milling, experimental parameters such as rotation speed, ball-milling time, ball-milling media, and the ball-to-powder mass ratio have an important influence on the ball-milled products even when using the same proportion and type of oxides. For example, Gajović reported that nanosized ZrTiO_4_ formed in ZrO_2_-TiO_2_ powder mixtures, whereas only amorphous mixtures were obtained during ball milling in Stubičar’s work [21,22]. In addition, the polymorphic transformation of Ln_2_O_3_ was also observed in Gd_2_O_3_-TiO_2_ and Y_2_O_3_-2TiO_2_ powder systems [23].

**Figure 2:**
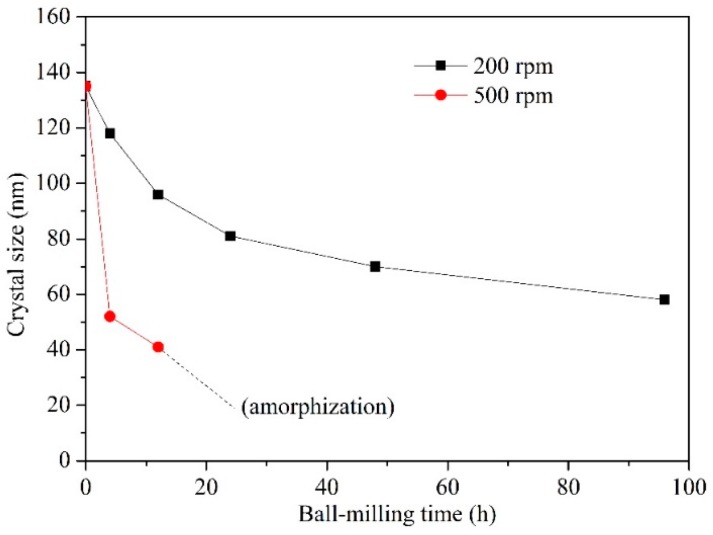
Curves of Dy_2_O_3_ grain size vs. ball-milling time.

The variation of Dy_2_O_3_ grain size with ball-milling time at the rotational speeds of 500 rpm and 200 rpm is shown in Figure 2. Actually, the size of Dy_2_O_3_ grain was calculated for Dy_2_O_3_ with cubic structure, not for Dy_2_O_3_ with monoclinic structure, because ball milling induced a phase transformation in Dy_2_O_3_ from cubic to monoclinic and simultaneously from monoclinic to amorphous. It is difficult to calculate the grain size of Dy_2_O_3_ with monoclinic structure. It can be seen that ball milling results in a fast decrease of Dy_2_O_3_ grain size in the initial stage at both 500 rpm and 200 rpm. The refinement rate of crystallite size is roughly logarithmic with ball-milling time at 200 rpm. After 96 h of ball milling, the size of Dy_2_O_3_ grain is up to approximately 60 nm. However, at 500 rpm, the diffraction peaks of Dy_2_O_3_ with cubic structure are hardly observed in the XRD spectrum after milling for 24 h as shown in Figure 1a, especially, when the ball-milling time is over 48 h. Therefore, the size of Dy_2_O_3_ with cubic structure is calculated only before 12 h of ball-milling time. It can be seen that the grain size is quickly decreased. In addition, after same ball-milling time, the grain size of Dy_2_O_3_ phase at the 500 rpm is obviously smaller than that at the 200 rpm. The effect of ball milling on the grain refinement of powder mixtures at 500 rpm is significantly more intense than that at 200 rpm. The size of Dy_2_O_3_ grain in the powder mixtures after milling for 4 h at 500 rpm is about 52 nm, which is smaller than that after milling for 96 h at 200 rpm (approximately 60 nm).

**Figure 3:**
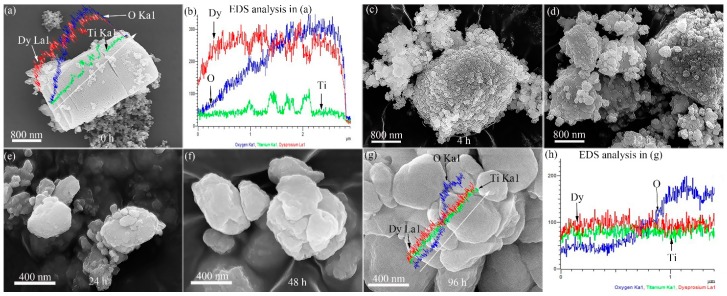
SEM analysis results of the powder mixtures milled at 500 rpm for different ball-milling times. The images showing the morphology of ball-milled mixtures for (**a**) 0 h; (**c**) 4 h; (**d**)12 h; (**e**) 24 h; (**f**) 48 h; and (**g**) 96 h; (**b**,**h**) are EDS analysis results of ball-milled particles in (**a**,**g**), respectively.

The morphology evolution of Dy_2_O_3_-TiO_2_ powder mixtures with increasing ball-milling time at 500 rpm is shown in Figure 3. Both TiO_2_ and Dy_2_O_3_ are brittle components, which are fragmented during ball milling and particle size reduces continuously as a consequence of the energy provided during ball milling. The morphology of large particles is changed significantly due to fracture, agglomeration, and deagglomeration processes. The morphology of the original powder mixtures consists of large-sized Dy_2_O_3_ particles in micrometer size and small-sized TiO_2_ particles in nanometer. The shape of the powder particles is irregular. The line-scanning results of elemental Dy, Ti, and O in the characteristic position in Figure 3a are shown in Figure 3b and also inserted in Figure 3a. It can be seen that the small-sized particles are TiO_2_ component and the large-sized particles are Dy_2_O_3_ components from the variation of the elemental diffraction intensity. The brittle Dy_2_O_3_ particles are fragmented by ball-powder-ball collisions, leading to a considerable reduction in the powder particle size and subsequent amorphization as milling time increases. After ball milling for 4 h, the size of particles decreases significantly. A large number of small size of Dy_2_O_3_ particles in nanometer can be observed in the milled powders as shown in Figure 3c. The morphology of the powder mixtures is transformed to uniform, as shown in Figure 3c–g, where the ball-milling time ranges from 4 to 96 h. The morphologies demonstrate that the refining effects of the powder particles are proportional to the ball-milling time for the same rotational speed. After 96 h of ball milling, a large number of nanoparticles agglomerate to form a large-sized particle, as shown in Figure 3g. In addition, TiO_2_ particles disappear after ball milling for 96 h, as shown in Figure 1a. The surfaces of the Dy_2_O_3_ particles in Figure 3g are clean compared with those in Figure 3a. It can be concluded that the particle size in the powder mixtures is refined to the nanoscale after ball milling for 96 h. The line-scanning results of elemental Dy, Ti, and O in the characteristic position in Figure 3g is shown in Figure 3h and also inserted in Figure 3g, which indicates these elements are uniformly distributed in the ball-milled particles according the variation of the elemental diffraction intensity. In addition, the morphology evolution of powder mixtures at an 200 rpm dose are not provided in the present work because the powder mixtures are only fined and homogenized according to the XRD results as shown in Figure 1b. The morphology of mixtures after ball milling for 96 h at 200 rpm is similar with that after ball milling for 4 h at 500 rpm.

**Figure 4:**
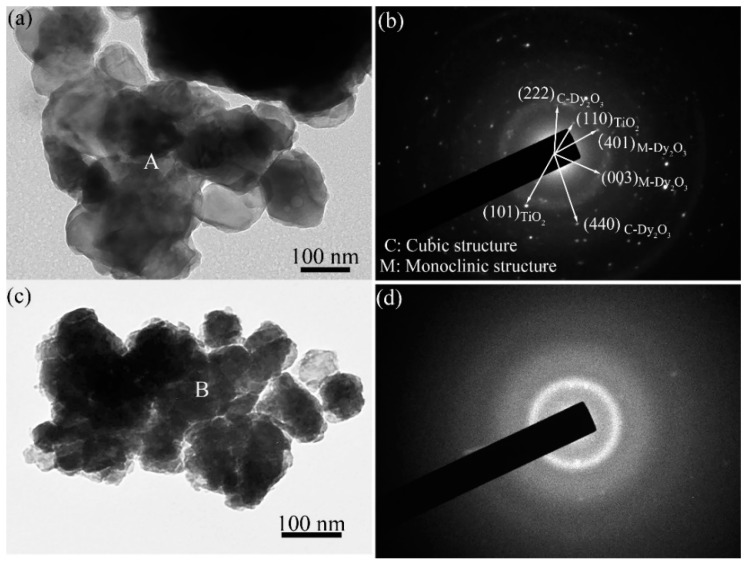
TEM analysis results of the mixtures ball milled at 500 rpm: (**a**) the bright field TEM image and (**b**) corresponding SAED pattern of mixtures milled for 4 h; (**c**) Bright field TEM image and (**d**) corresponding SAED pattern of mixtures milled for 96 h.

Figure 4 shows TEM images and corresponding selected area electron diffraction (SAED) patterns of Dy_2_O_3_-TiO_2_ powder mixtures milled for 4 h and 96 h. After milling for 4 h, nano-sized ball-milled powder particles aggregate to form large-sized particles, as shown in Figure 4a, due to the high active surface energy created upon ball milling. The size of the original TiO_2_ particles is approximately 50 nm. From the XRD results, it can be observed that ball milling leads to TiO_2_ particle refinement, dissolution, and finally disappearance after 96 h. Therefore, the main particles presented in the TEM image are Dy_2_O_3_. The bright zones near the edge of the powder particles are the thin areas where the electron beam penetrates. The dark areas in the images of the powder particles are the thick areas where the electron beam rarely penetrates. Indexing and analyzing the ring-shaped SAED pattern taken from the area denoted by the letter “A” indicates that the Dy_2_O_3_ grains are already nanocrystalline. The diffraction spots coming from cubic Dy_2_O_3_, monoclinic Dy_2_O_3_, and TiO_2_ grains are present in the SAED pattern in Figure 4b. The diffraction halo in the SAED pattern also indicates the formation of an amorphous phase during high-energy ball milling.

After ball milling for 96 h, the ball-milled powders agglomerate to form large particles with large thicknesses such that the electron beam rarely penetrates, showing as a dark color in the TEM image in Figure 4c. It is difficult to observe the microstructure of the agglomerated particles. The SAED pattern from the area marked with the letter “B” indicates that the powder mixtures are almost completely converted to the amorphous phase, although sporadic diffraction spots are also present in this pattern. The atom arrangement in the amorphous phase is disordered over long distances, but ordered over short distances. As grain size decreases, the number of atoms at the grain boundaries increases. The proportion of atoms in the crystal volume relative to the crystal boundary decreases. Schwarz and Koch noted that the formation of an amorphous phase in as-milled powders was similar to the amorphization that occurs during the isothermal annealing of crystalline metallic thin films [24]. In high-energy ball milling, the intense deformation accelerates interdiffusion, and the large defect density increases the free energy of the components in the mixture to form an amorphous product.

**Figure 5:**
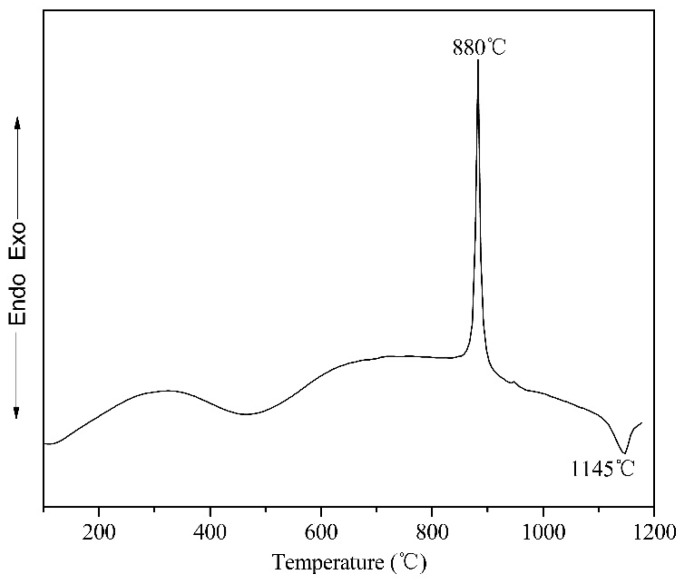
DSC curve of Dy_2_O_3_-TiO_2_ powder mixtures milled for 96 h.

Monoclinic Ln_2_O_3_ is initially formed in the mechanical alloying of lanthanum titanate or dititanate. In Moreno’s research, the formation of Gd_2_(Ti_(1−*y*)_Zr*_y_*)_2_O_7_ pyrochlores occurred in the final step of ball milling starting from an amorphous matrix of Gd_2_O_3_, TiO_2_, and ZrO_2_ [23]. However, in the present work, after 96 h of ball milling, the monoclinic Dy_2_O_3_ phase could not be detected and was completely transformed to the amorphous phase. Moreover, dysprosium titanate also could not be detected. To investigate the sintering behavior of the ball-milled powder mixtures, DSC was carried out on the powder mixtures milled for 96 h at test temperatures ranging from 200 to 1200 °C. The DSC curve in Figure 5 shows one exothermic peak close to 880 °C and one endothermic peak close to 1145 °C. An exothermic peak in a DSC curve can generally be attributed to a transition from disordered to ordered, the recrystallization of original components from the amorphous phase or the formation of a new compound from the ball-milled amorphous powders. Therefore, subsequent X-ray analysis of the ball-milled powders annealed at different temperatures is used to further analyze occurrences in the heating process in more detail. The powder mixtures transform completely to the amorphous state after ball milling for 96 h. Therefore, it seems feasible that the transition from disordered to ordered produced the exothermic peak in the DSC curve. In fact, only the amorphous peak is observed in the XRD pattern of the 96 h ball-milled powder after annealing for 24 h at 700 °C. No diffraction peaks for Dy_2_O_3_ or TiO_2_ are detected. Even with prolonged annealing time, the XRD results are the same. Therefore, the exothermic peak in the DSC curve should be related to the new phase generated from the amorphous mixtures.

**Figure 6:**
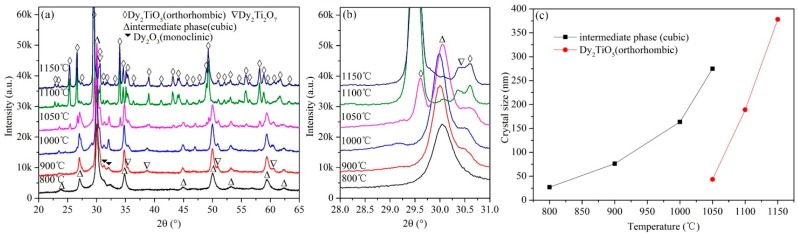
XRD patterns of the ball-milled powder mixtures annealed for 3 h at various temperatures at diffraction angles 2θ ranging from (**a**) 20° to 65° and (**b**) 28° to 31°; (**c**) Curves of the grain size of main characteristic phase vs. annealing temperature. The powder mixtures were previously milled for 96 h at 500 rpm.

The powder mixtures milled for 96 h were annealed for 3 h at 800, 900, 1000, 1050, 1100, and 1150 °C. The XRD results of the annealed powder mixtures are shown in Figure 6a,b. Several diffraction peaks different from the diffraction peaks of Dy_2_O_3_ (cubic and monoclinic crystal structure) and TiO_2_ (rutile structure) are observed, which indicates new components with crystal structures generated from the amorphous mixtures. This experimental phenomenon of synthesizing new compounds is similar to Stubičar’s research in which the orthorhombic ZrTiO_4_ phase was generated from the high-temperature annealing of amorphous mixtures formed from the ball milling of a ZrO_2_-TiO_2_ powder system [21] and consistent with Khor’s results that zirconia was produced from annealing amorphous mixtures formed from the ball milling of an equimolar ZrSiO_4_ and Al_2_O_3_ powder system [25].

According to the XRD standard database, cubic Dy_2_TiO_5_ {111} presents at 2θ = 30.043°, hexagonal Dy_2_TiO_5_ {102} presents at 2θ = 32.411°, orthorhombic Dy_2_TiO_5_ {201} presents at 2θ = 29.554°, and pyrochlore Dy_2_Ti_2_O_7_ {111} presents at 2θ = 30.698°; the difference in the above diffraction angles is not very large. Three diffraction peaks are observed in the XRD patterns of the powder mixtures annealed for 3 h at 800, 900, and 1000 °C. The main diffraction peak representing the crystalline phase is at 2θ = 30.0°, as shown in Figure 6b. Therefore, the newly formed product in the powder system annealed between 800 °C and 1000 °C is not pyrochlore Dy_2_Ti_2_O_7_. This result is different than that found in Amit Sinha’s research in which pyrochlore Dy_2_Ti_2_O_7_ was initially created in the formation of Dy_2_TiO_5_ [19]. In their research using an equimolar Dy_2_O_3_-TiO_2_ system, the chemical reaction of Dy_2_O_3_ and TiO_2_ initially formed pyrochlore Dy_2_Ti_2_O_7_, and then Dy_2_Ti_2_O_7_ reacted with the remaining Dy_2_O_3_ to form orthorhombic Dy_2_TiO_5_.

According to the equilibrium phase diagram, the Dy_2_TiO_5_ phase has three crystal structure types depending on the temperature: orthorhombic $\overset{1350\;{}^{\circ}\text{C}}{↔}$ hexagonal $\overset{1680\;{}^{\circ}\text{C}}{↔}$ cubic [15]. Transformation from the high-temperature phase to the low-temperature phase is an exothermic process. For example, the polymorphic transformation of Gd_2_TiO_5_ from hexagonal to orthorhombic produced an exothermic peak in the DSC curve [7]. However, in the present work, there is only one exothermic peak in the DSC curve. Although the diffraction peaks in the XRD patterns match well with the diffraction peaks in the standard pattern of cubic Dy_2_TiO_5_, the generated phase should not be cubic Dy_2_TiO_5_ because low-temperature phases of orthorhombic and hexagonal Dy_2_TiO_5_ were not detected after annealing over temperatures ranging from 700 to 1000 °C for annealing times ranging from several minutes to 3 h; additionally, as mentioned above, the formation temperature of cubic Dy_2_TiO_5_ is over 1680 °C. It is not possible to achieve such a high temperature during ball milling. Therefore, the high-temperature phase of cubic Dy_2_TiO_5_ should not be produced. Instead, the generated phase is an intermediate phase that has a similar crystal structure to cubic Dy_2_TiO_5_ and is a metastable state that phase transforms to orthorhombic Dy_2_TiO_5_ when the annealing temperature is above 1050 °C. After annealing for 3 h at 1100 °C, orthorhombic Dy_2_TiO_5_ and a small amount of pyrochlore Dy_2_Ti_2_O_7_ and cubic Dy_2_O_3_ are detected; notably, cubic Dy_2_TiO_5_ is not observed in the ball-milled powder mixtures.

The change of the grain size of the main characteristic phase in the annealed powder mixtures with annealing temperature is shown in Figure 6c. According to the above results, the grain size of the intermediate phase and orthorhombic Dy_2_TiO_5_ are calculated using Suryanarayana and Grant Norton’s formula. It can be seen that the grain size increases with increasing annealing temperature. The grain size of the intermediate phase is about 27 nm in the powder mixtures annealed for 3 h at 800 °C and is about 275 nm in the powder mixtures annealed for 3 h at 1050 °C. Because the intermediate phase transforms to orthorhombic Dy_2_TiO_5_ when the annealing temperature is above 1050 °C, the grain size of orthorhombic Dy_2_TiO_5_ is calculated at the annealing temperature ranging from 1050 °C to 1150 °C. The grain size of orthorhombic Dy_2_TiO_5_ is about 43 and 380 nm after annealing for 3 h at 1050 °C and 1150 °C, respectively.

In addition, the pressure created during ball milling is not high enough to transform the crystal structure of Dy_2_TiO_5_ from orthorhombic to hexagonal or from hexagonal to cubic. The average pressure on the contact surface of two colliding mill balls is approximately 8.5 GPa [26], which is far below the 100 GPa needed to induce a pressure wave to cause the Gd_2_TiO_5_ phase transformation from the low-temperature orthorhombic phase to the high-temperature hexagonal phase [27]. Therefore, the intermediate phase is not produced by collision pressure. Further investigation is needed into the cause of the formation of the intermediate phase.

**Figure 7:**
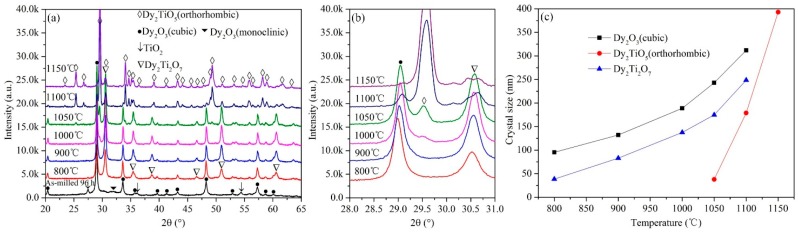
XRD patterns of the ball-milled powder mixtures annealed for 3 h at various temperatures at diffraction angles 2θ ranging from (**a**) 20° to 65° and (**b**) 28° to 31.0°; (**c**) Curves of the grain size of characteristic phase vs. annealing temperature. The powder mixtures were previously milled for 96 h at 200 rpm.

To further investigate the annealing behavior of the ball-milled powder mixtures, the powder mixtures milled for 96 h at 200 rpm were sintered for 3 h at 800, 900, 1000, 1050, 1100, and 1150 °C. The phase evolution of the annealed powder mixtures identified by XRD analysis is shown in Figure 7. Under these ball-milling conditions, the Dy_2_O_3_-TiO_2_ powder mixtures are homogenized, and the polymorphic transformation of Dy_2_O_3_ from cubic to monoclinic is not observed in Figure 7a. In the XRD pattern of the powder mixtures annealed for 3 h at 1000 °C, diffraction peaks for Dy_2_O_3_ and Dy_2_Ti_2_O_7_ phase are observed. However, the main diffraction peak for orthorhombic Dy_2_TiO_5_ is not detected. Under these conditions, the annealed powder mixtures are composed of cubic Dy_2_O_3_ and pyrochlore Dy_2_Ti_2_O_7_. The powder mixtures generate the orthorhombic Dy_2_TiO_5_ phase at 1050 °C and are composed of cubic Dy_2_O_3_, pyrochlore Dy_2_Ti_2_O_7_, and orthorhombic Dy_2_TiO_5_. The powder mixtures are almost completely transformed to orthorhombic Dy_2_TiO_5_ after annealing at 1150 °C for 3 h. The diffraction peak intensity of orthorhombic Dy_2_TiO_5_ gradually increases with increasing annealing temperature, and simultaneously, the diffraction peak intensity of Dy_2_O_3_ and Dy_2_Ti_2_O_7_ decreases with increasing annealing temperature. For powder mixtures ball milled for 96 h at 200 rpm, the evolutionary behavior at various annealing temperatures is consistent with the data presented in Ref. [19], in which Amit Sinha reported that the chemical reaction of Dy_2_O_3_ and TiO_2_ initially formed pyrochlore Dy_2_Ti_2_O_7_, and then Dy_2_Ti_2_O_7_ reacted with the remaining Dy_2_O_3_ to form orthorhombic Dy_2_TiO_5_. However, this experimental phenomenon is different from that observed in the annealed powder mixtures milled for 96 h at 500 rpm, as shown in Figure 6, due to the initial conditions of the ball-milling mixtures.

The change of the grain size of main characteristic phase in the annealed powder mixtures with annealing temperature is shown in Figure 7c. The grain size of the cubic Dy_2_O_3_, pyrochlore Dy_2_Ti_2_O_7_ and orthorhombic Dy_2_TiO_5_ are calculated using Suryanarayana and Grant Norton’s formula. The grain size of cubic Dy_2_O_3_ and pyrochlore Dy_2_Ti_2_O_7_ increases with increasing annealing temperature. Because the orthorhombic Dy_2_TiO_5_ is detected in the powder mixtures annealed at 1050 °C for 3 h, the grain size of orthorhombic Dy_2_TiO_5_ is calculated when the annealing temperature is over 1050 °C. The grain size of orthorhombic Dy_2_TiO_5_ is about 38 nm and 395 nm after annealing 3 h at 1050 °C and 1150 °C, respectively.

**Figure 8:**
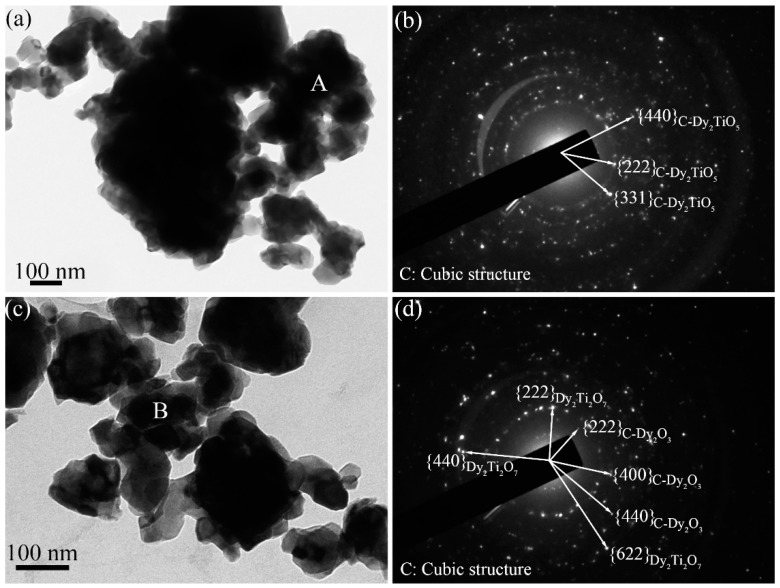
The bright field TEM images and corresponding SAED patterns of the ball-milled Dy_2_O_3_-TiO_2_ powder mixtures annealed at 1000 °C for 3 h, (**a**,**b**) the powder mixtures are previously milled for 96 h at 500 rpm; (**c**,**d**) the powder mixtures are previously milled for 96 h at 200 rpm.

Figure 8a,b shows the bright field TEM image and corresponding SAED pattern of the ball-milled Dy_2_O_3_-TiO_2_ powder mixtures annealed at 1000 °C for 3 h, respectively. The powder mixtures are previously milled for 96 h at 500 rpm. After annealing for 3 h, the grain size of powder mixtures is kept in nanometer scale, which can be supported by the corresponding SAED pattern taken from the region marked letter “A” in Figure 8a. The diffraction ring is a typical SAED pattern of nanocrystal materials. After analyzing and indexing the ring-shaped SAED pattern, it is indicated that this SAED pattern belongs to the intermediate Dy_2_TiO_5_ phase that has a similar crystal structure to cubic Dy_2_TiO_5_, which is in accord with the XRD results as shown in Figure 6. The small-sized powders agglomerate to form large particles with large thicknesses as shown in Figure 8a. The bright zones near the edge of the powder particles is the thin area where the electron beam penetrates. The dark area in the image of the powder particles is the thick area where the electron beam rarely penetrates. After annealing, the amorphous ball-milled powder mixtures are changed to the intermediate Dy_2_TiO_5_ phase with crystal structure. Figure 8c,d is the bright field TEM image and corresponding SAED pattern of the annealed Dy_2_O_3_-TiO_2_ powder mixtures that were previously milled for 96 h at 200 rpm. Indexing and analyzing the ring-shaped SAED pattern taken from the area denoted by the letter “B” indicates that the particles are composed of cubic Dy_2_O_3_ and pyrochlore Dy_2_Ti_2_O_7_, which is accord with the XRD results of the powder mixtures annealed at 1000 °C for 3 h as shown in Figure 7. This experimental result is different from that observed in the annealed powder mixtures milled for 96 h at 500 rpm.

## 4. Conclusions

The microstructural evolution of Dy_2_O_3_-TiO_2_ powder mixtures during ball milling and post-milled annealing was investigated using TEM, SEM, XRD, and DSC. The conclusions can be made as follows:

1. The ball-milling parameters had a great effect on ball milling and the subsequent annealing process.
2. At 500 rpm rotation speeds, the mixtures were fined, homogenized, nanocrystallized, and then completely amorphized, and the crystal structure of Dy_2_O_3_ was transformed from cubic to monoclinic. The amorphous transformation resulted from monoclinic Dy_2_O_3_, not from cubic Dy_2_O_3_. However, at 200 rpm rotation speeds, the Dy_2_O_3_-TiO_2_ powder mixtures were only homogenized, and the polymorphic transformation of Dy_2_O_3_ from cubic to monoclinic was not observed. Meanwhile, the powder mixtures did not transform to the amorphous phase.
3. The powder mixtures milled for 96 h at 500 rpm were annealed for 3 h at a temperature range of 800 to 1000 °C. An intermediate phase with a crystal structure similar to that of cubic Dy_2_TiO_5_ was synthesized, which was a metastable phase that transformed to orthorhombic Dy_2_TiO_5_ when the annealing temperature was above 1050 °C. However, the powder mixtures milled for 96 h at 200 rpm did not transform to the amorphous phase. The annealing behavior showed that the chemical reaction of Dy_2_O_3_ with TiO_2_ initially formed pyrochlore Dy_2_Ti_2_O_7_, and then Dy_2_Ti_2_O_7_ reacted with the remaining Dy_2_O_3_ to form orthorhombic Dy_2_TiO_5_.
